# In-vitro effect of different instrumentations on adhesion of a multispecies bacterial mixture to root surfaces and the subsequent response of pulpal cells

**DOI:** 10.1007/s00784-025-06321-5

**Published:** 2025-04-24

**Authors:** Alexandra Stähli, Sarah Feuz, Anja Elisabeth Jutzi, Luciana Pisenti Berto, Sandor Nietzsche, Anton Sculean, Sigrun Eick

**Affiliations:** 1 Department of Periodontology, School of Dental Medicine, University of Bern, Bern, Switzerland; 2 Department of Restorative, Preventive and Pediatric Dentistry, School of Dental Medicine, University of Bern, Bern, Switzerland; 3 Center for Electron Microscopy, Jena University Hospital, Jena, Germany

**Keywords:** Periodontal therapy, Exposed root surface, Bacterial penetration, Immune response of pulpal cells

## Abstract

**Objectives:**

To investigate in vitro the influence of instrumentation methods on the bacterial colonization of root dentine and pulpal cell behaviour seeded into the pulp cavum.

**Materials and methods:**

Extracted teeth underwent root canal treatment with sealing of apices. The teeth were subjected to periodontal instrumentation using manual, ultrasonic scalers, air polishing, or left untreated. The root surfaces were then incubated with a mixture of six bacterial species for 2 h, 24 h and 10 weeks, before dentine samples were taken and analyzed for bacterial colony forming units (cfu) counts. In an additional series, pulpal cells were seeded into the reopened pulp chambers after 10 weeks of incubation with the bacterial mixture and analysed for interleukin (IL)-8 and matrix metalloprotease (MMP)-3 expression.

**Results:**

After 2 and 24 h of incubation, instrumented dentine samples contained fewer bacteria than the controls (median (log10 cfu): 5.73 vs. 5.92; 7.71 vs. 8.01 (*p* = 0.007; *p* = 0.017)). At 24 h, among the instrumentation groups the highest cfu counts were observed in the ultrasonic group (*p* = 0.012 vs. manual scaler group; *p* = 0.002 vs. air polishing group). After 10 weeks, the number of viable bacteria (cfu) decreased in all groups with no difference between any group. Pulpal cells seeded in teeth, with or without prior instrumentation, but exposed to the bacterial mixture for 10 weeks, released higher levels of IL-8 and MMP-3 compared to those in uncontaminated and untreated controls.

**Conclusions:**

Instrumentation initially inhibits bacterial colonisation. Prolonged exposure of the outer root surface to bacteria may increase the inflammatory response of pulpal cells.

**Clinical relevance:**

Regular removal of bacteria from the root surface is supported by these in vitro data.

## Introduction

Periodontal disease is highly prevalent, affecting 61.6% of the general population. This rate rises to nearly 80% among individuals aged 65 and older, underscoring age as a significant contributing factor [1]. Periodontitis is linked to a dysbiotic biofilm microbiota and a disrupted host immune response [2, 3]. Mechanical removal of biofilms remains the gold standard for prevention and therapy. The European Federation of Periodontology (EFP) S3 level clinical practice guideline strongly recommends subgingival instrumentation, which effectively reduces the number of diseased sites in the second step of therapy, known as cause-related therapy [4]. According to the guideline, both manual and power-assisted sonic or ultrasonic instruments are suitable for this purpose [4]. To maintain periodontal stability, supportive periodontal care is recommended at regular intervals of 3 to 12 months [4]. Professional mechanical plaque removal is recommended as an integral part of this supportive care [4].

Endodontic-periodontal lesions represent a pathological communication between the pulpal and periodontal tissues of a tooth [5]. These lesions can be triggered by, among other factors, periodontal destruction that affects pulp viability [5].

As the tooth is a single biological structure, therapeutic interventions such as subgingival instrumentation can have "far-reaching" effects on the pulp and vice versa [6]. Extensive or repeated instrumentation can alter the root-cementum surface [7] affecting bacterial adhesion [7], penetration and the health of the pulpal tissue [8]. Connections between the periodontium and pulpal tissues occur primarily through exposed dentinal tubules, lateral or accessory canals and the apical foramen. When dentinal tubules are exposed, microorganisms can migrate in both directions [9, 10]. It remains unclear how different mechanical instrumentation methods, such as conventional scaling and root planing, ultrasonic scaling and air-powder polishing, affect the interaction between periodontal bacteria and pulpal tissues.

It is well known, that dental instruments not only remove biofilm and calculus, but also cause loss of apatite from root surfaces, which increases with instrumentation time [11]. In our own in vitro study, we quantified the thickness loss of standardised dentine specimens. A single cycle of manual instrumentation resulted in a mean loss of dentine of 21 μm, which was greater than the loss observed after ultrasonic or air polishing. In addition, manual instrumentation produced a rougher surface compared to the other treatments [12].

The question therefore arose as to whether instrumentation, which clearly damages the root surfaces, promotes bacterial penetration from the subgingival biofilm through the dentine to the pulp. Recently, we demonstrated that scaling increased the number of samples showing bacterial penetration after 10 weeks [13]. Scanning electron microscopy images confirmed the presence of bacteria in the irregularities of the dentine close to the scaled root surface [13].

The aim of the present study was to investigate the effect of different mechanical instrumentation modalities on adhesion and penetration of a periodontitis-associated bacteria mixture into dentine, as well as the cellular response of pulpal cells seeded into the pulp cavities of extracted, instrumented and non-instrumented teeth exposed to these bacteria.

The effect of instrumentation was compared with non-treated teeth and the different treatment modalities were specifically analysed. The study addressed the following questions: i) what is the effect of instrumentation on bacterial colonisation, ii) what is the cellular response of pulpal cells (fibroblasts) seeded in the pulp cavities after exposure of the teeth to a 10-week-old biofilm, iii) and are there differences between the instrumentation modalities?

## Material and methods

**Fig. 1:**
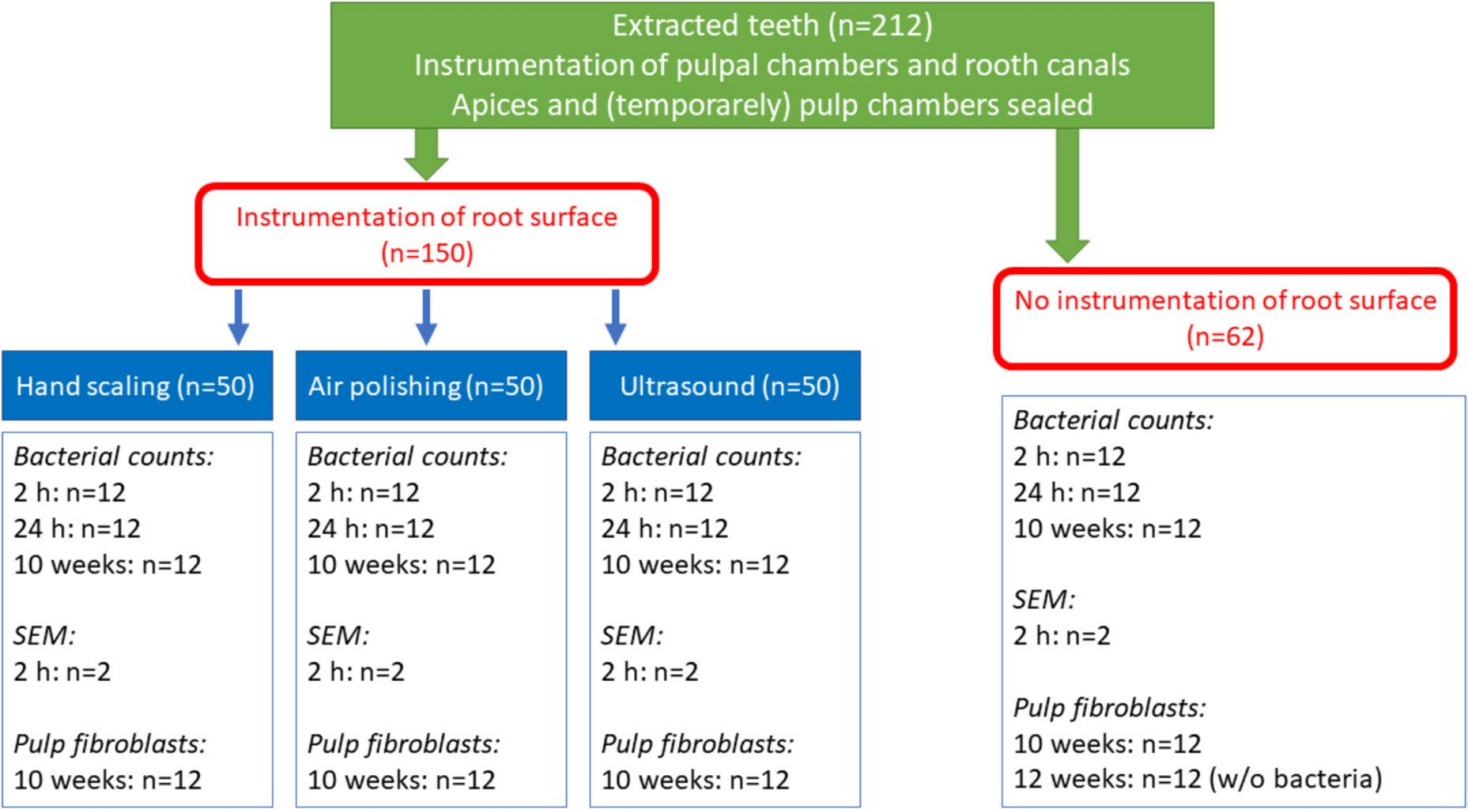
Flowchart of the different groups of the teeth used, the experimental design including the incubation times in the presence of a 6-species mixture (only exception: one group had been incubated for 10 weeks in nutrient broth w/o bacteria)

The study protocol is shown in Fig. 1, detailing the use of teeth as well as the different steps.

### Teeth preparation

A total of 212 extracted multi-rooted teeth, free of visible carious lesions and high amounts of calculus, were prepared for the experiments. Since the teeth were classified as "irreversibly anonymised" according to the guidelines, prior approval from the Cantonal Ethics Committee (KEK) was not required.

 The pulpal chambers and root canals were prepared with endodontic K-files (#10–40), instrumented with WaveOne Gold and negotiated up to the medium or large instruments, depending on the gauging of the apical constriction. Master gutta-percha cones of primary size (25/0.07), medium size (35/0.06) or large size (40/0.05) were fitted. During root canal preparation, the teeth were rinsed with a 0.5% NaOCl solution. To prevent apical leakage, the root canal apices were sealed with composite (UltraEtch, MS Dental AG, Busswil, Switzerland, Optibond FL, Kerr Dental, Kloten, Switzerland, and Ceram X, Dentsply Sirona AG, Baden, Switzerland). In addition, the outer apical third of each tooth was covered with composite, while the coronal pulp chamber was filled with a cotton pellet and thereafter closed with a provisional material (Telio Inlay Temporary Filling, Ivoclar Vivadent, Schaan, Liechtenstein). Then, after placing the teeth in water, they were sterilised at 121 °C for 10 min and subsequently maintained under sterile conditions. Since extracted teeth are often exposed to antimicrobials during the treatment before extraction, what could negatively affect microbial colonization, and to remove potential compounds released from the added composite, the teeth were stored in a 0.9% w/v NaCl solution for one month, with an exchange of the solution every 3 days.

### Instrumentation of teeth

The teeth underwent one of the following three treatments by experienced clinicians (A.St and L.A.B): i) scaling and root planing with Gracey curettes (Deppeler SA, Rolle, Switzerland) for 15 strokes per side, ii) ultrasonic treatment for 15 s per side (Airflow Prophylaxis Master, PiezonLed, EMS, Nyon, Switzerland), or iii) air polishing with erythritol (Airflow Prophylaxis Master, airpolishing max, EMS, Nyon, Switzerland) for 15 s per side (with removal of visible calculus by ultrasonic scaling beforehand), or were left untreated. After treatment, the teeth were placed in a 0.9% w/v NaCl solution for at least one week which was exchanged after 3 days. The sterility of the solution was tested before exposing the teeth to bacterial mixture. For this purpose aliquots of 1 ml were given into 9 ml of nutrient broth and incubated at 37 °C for 48 h.

### Microorganisms

The 6-species mixture included *Streptococcus gordonii* ATCC 10558, *Actinomyces oris* MG1, *Fusobacterium nucleatum* ATCC 25886, *Parvimonas micra* ATCC 33270, *Tannerella forsythia* ATCC 43037 and *Porphyromonas gingivalis* ATCC 33277. These strains were pre-cultured on trypticase soy agar (TSA) plates (Oxoid, Basingstoke, GB) with 5% sheep blood for 24 h at 37 °C under anaerobic conditions (*S. gordonii* with 10% CO_2_). The microorganisms were then suspended in a 0.9% w/v sodium chloride (NaCl) solution to an optical density of OD600 nm = 1. The suspensions were then mixed in the following proportions 1 part *S. gordonii*, 2 parts *A. oris* and 4 parts each of the other species. (Using this mixture guarantees a balanced regrowth of all included species as checked in preliminary experiments.) The mixture was added to the nutrient broth (Wilkins-Chalgren broth with 10 µg/ml β-NAD) in a ratio of 1:100.

### Short-term experiments

The instrumented teeth were placed in holders, ensuring that the upper half of the crown was fixed in a mesh. The holder was then transferred to a corresponding box so that the roots and lower parts of the crowns were immersed in nutrient broth containing the bacterial mixture. After incubation in an anaerobic atmosphere at 37 °C for either 2 or 24 h, the bacterial load in the superficial dentine was quantified.

Teeth were taken out of the holder and the biofilms on the outer parts of the roots were removed by intensive wiping with a cotton swab, before teeth were shortly (10 s) bathed in a 3% hydrogen peroxide solution. Then, dentine samples were collected using a 2.5 mm rose burr. The burr was drilled until its head reached the level of the tooth surface, with continuous cooling throughout the process. The collected dentine samples (approximately 8 mm^3^ volume) were placed in 0.9% w/v NaCl solution. The samples were subsequently processed by vortexing, ultrasonication, and pipetting, followed by the preparation of a tenfold dilution series. Aliquots from each dilution were then plated onto agar plates. After an anaerobic incubation for 10 days, the colony forming units (cfu) were counted.

To visualize teeth surfaces, the SEM group of teeth were handled as described above but without exposing the surface to disinfection. Instead of taking dentine samples, the teeth were placed in tubes containing 0.1 M cacodylate buffer and immediately sent to the Centre for Electron Microscopy at the University Hospital of Jena. There, after additional washing with 0.1 M cacodylate buffer, the samples were sequentially dehydrated in increasing concentrations of ethanol (30%, 50%, 70%, 90% and 100%) for 10 min each. The samples were then critical point dried with liquid CO_2_ and sputter coated with platinum (approximately 1 nm thick) using an SCD005 sputter coater (BAL-TEC, Liechtenstein) to prevent surface charging. Finally, the samples were examined using a LEO-1530 field emission (FE) scanning electron microscope (Carl Zeiss NTS GmbH, Oberkochen, Germany).

### Long-term experiments

The protocol for the long-term experiments was similar to that for the short-term experiments, but with extended incubation times. Extracted and instrumented teeth were incubated in nutrient media with bacteria for 10 weeks. The medium was changed twice a week, checked for contamination and bacteria were added if necessary. At the 10-week mark, cfu counts were analysed in the same way as for the 2-h and 24-h incubations.

In addition, two separate series were performed. Teeth were instrumented and exposed to bacteria for 10 weeks, as described above. Here, an additional control group, without instrumentation, was included and exposed to nutrient broth without bacteria for 10 weeks. After the 10-week period, the pulp chambers of the teeth were carefully opened, coated with 0.1% gelatine and seeded with pulpal cells. This procedure was recently published [5]. In short, pulpal cells were obtained from extracted donor teeth of 3 patients who had given informed and written consent for their use in research. As before, the teeth were classified as "irreversibly anonymised" according to the guidelines, no prior approval from the Cantonal Ethics Committee (KEK) was required. A total of three pulp cell strains were established by explant culture, with fewer than 10 passages used for the experiments. Cells were expanded to a concentration of 300,000 cells/ml in cell culture medium (DMEM, Invitrogen; Carlsbad, CA, USA) containing 1% fetal bovine serum (FBS, Invitrogen) and 20 µl (about 6000 cells) were pipetted per pulp cavum. Morphology of the dental pulp cells suggested mainly fibroblasts. After 48 h of incubation with 5% CO_2_, the cell culture medium was collected and immediately stored at −80 °C until analysis. Within 14 days, IL-8 and MMP-3 levels were quantified using commercially available ELISA kits (DuoSet® ELISA Development Systems kits, R&D Systems, Inc., Minneapolis, Minnesota, USA) according to the manufacturer's instructions. The detection limits were 20 pg/ml for MMP-3 and 350 pg/ml for IL-8.

### Statistics

All of the results were obtained in two independent runs. Per experiment 6 independent results (in a total 12 results), were subjected to statistical analysis using SPSS 29.0 (IBM, Chicago, IL, USA). Based on data of an in-vitro study on instrumentation a difference of 0.4 log10 is assumed between the instrumentation groups and control [12] this would result in 20 samples for each of the treatment groups (manual, ultrasonic scalers, air polishing; here we had n=50 per group) and 7 for the control group needed.

Due to the lack of a normal distribution, non-parametric tests were used. The Mann–Whitney test was used for comparisons between two groups, and the Kruskal–Wallis test for comparisons between more than two groups. For post-hoc comparisons between two groups after the Kruskal–Wallis test, the Mann–Whitney test with Bonferroni correction was used. The level of statistical significance was set at a *p*-value of 0.05.

## Results

### Colony forming unit (cfu) counts in dentine samples 2 h and 24 h after instrumentation

**Fig. 2:**
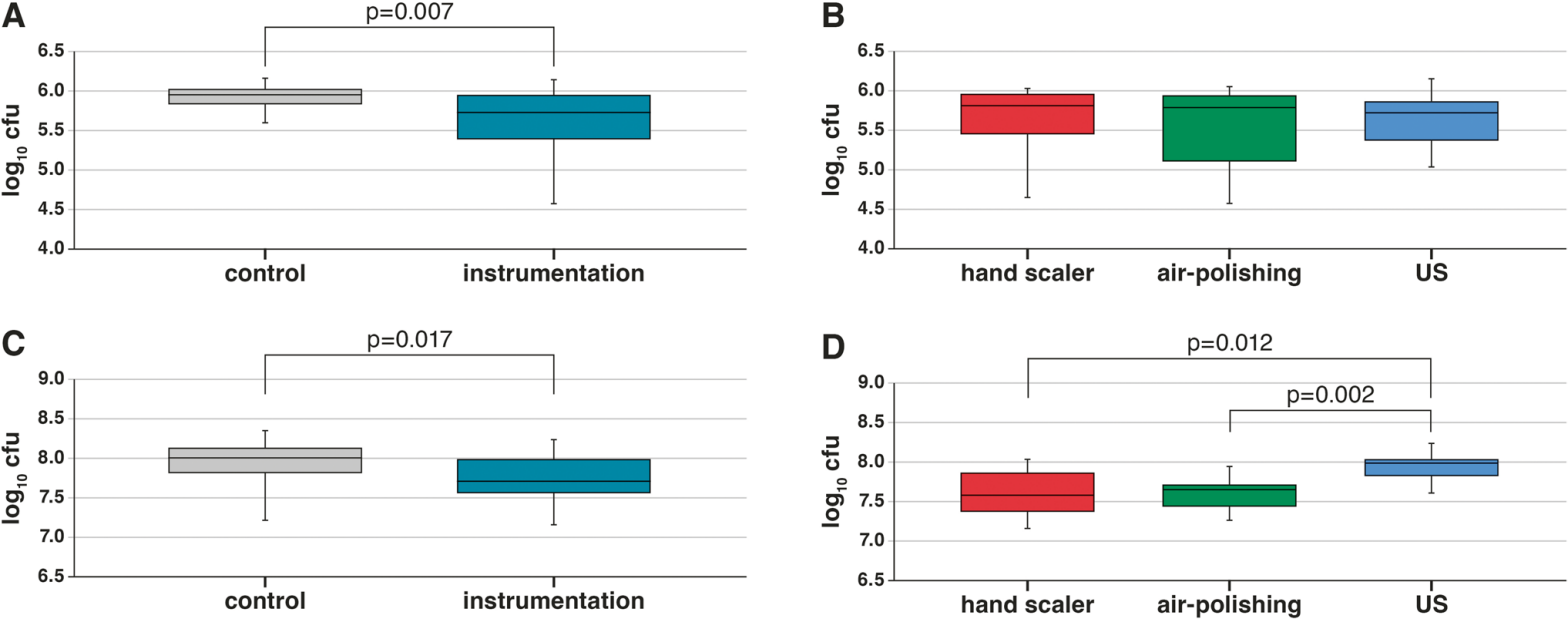
Colony forming unit (cfu) counts in dentine samples of the instrumented vs. non-instrumented surface (control) (**A**, **C**), after different instrumentations (**B**, **D**) and after subsequent incubation with a 6-species mixture for 2 h (**A**, **B**) and 24 h (**C**, **D**)

After 2 h of incubation, the dentine control samples contained a median of 5.97 log10 cfu bacteria, while instrumented samples had a median of 5.73 log10 cfu (total counts reduction vs. control by 42.5%). The difference is statistically significant (*p* = 0.007; Fig. 2A). Among the instrumentation groups there were only minor differences which did not reach statistical significance (Fig. 2B).

Twenty-four hours after instrumentation 7.71 log10 were counted, the differences remained statistically significant compared to the control group, which had a median of 8.01 log10 cfu (*p* = 0.017; Fig. 2C). Among the instrumentation groups most cfu were counted in the ultrasonic group which was statistically significantly higher than in the manual scaler group (*p* = 0.012) and in the air polishing group (*p* = 0.002; Fig. 2D).

### Scanning electron microscopy photographs

**Fig. 3:**
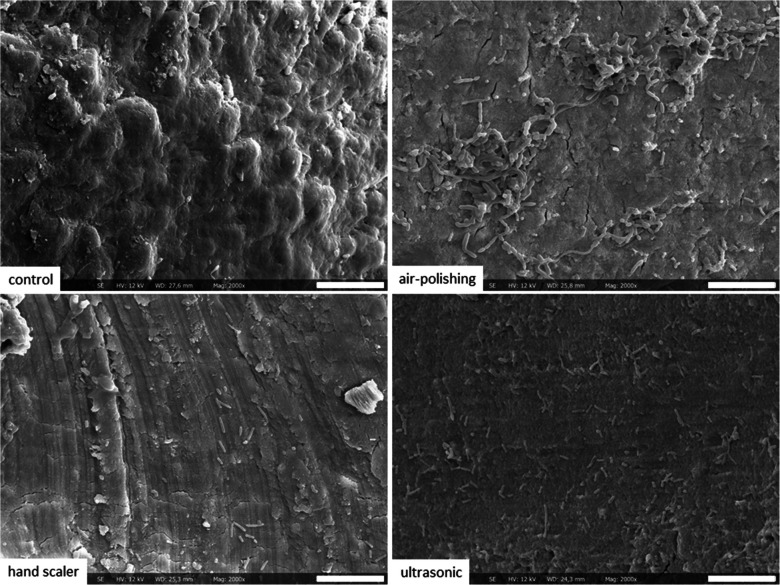
SEM photographs of the root surfaces below the cementum-enamel junction without treatment (top left) and after receiving instrumentation with a manual scaler (down left), with air polishing (top right) or with an ultrasonic scaler (down right) and subsequent cultivation with a 6-species mixture for 24 h (400-fold magnification, bar 60 μm)

SEM photographs were taken to examine the different surfaces. The untreated control remained covered by the cementum layer, which was in part removed in all treatment groups. Manual scaling resulted in noticeable scratches, whereas root dentine treated by air polishing and ultrasonics showed a very smooth surface (Fig. 3).

### Long-term incubation of instrumented teeth with bacteria

**Fig. 4:**
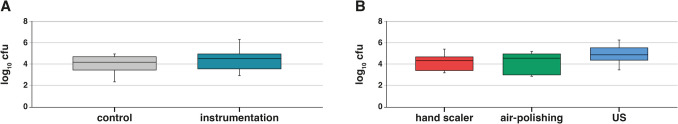
Colony forming unit (cfu) counts in dentine samples of the instrumented vs. non-instrumented surface (control) (**A**), after different instrumentations (**B**) upon 10 weeks of subsequent incubation with a 6-species bacterial mixture

In the long-term experiments, extracted and instrumented teeth were incubated in culture media with bacteria for 10 weeks. The cfu counts were lower than those observed in the short-term experiments. In the control group, dentine samples contained a median of 4.15 log10 cfu. Although the cfu counts tended to be higher in the instrumented groups (median 4.56 log10), these differences did not reach statistical significance (Fig. 4A). Among the instrumented groups, the ultrasonic group had the highest cfu counts with a median of 4.86 log10 cfu, but there was no statistically significant difference between the groups (Fig. 4B).

**Fig. 5:**
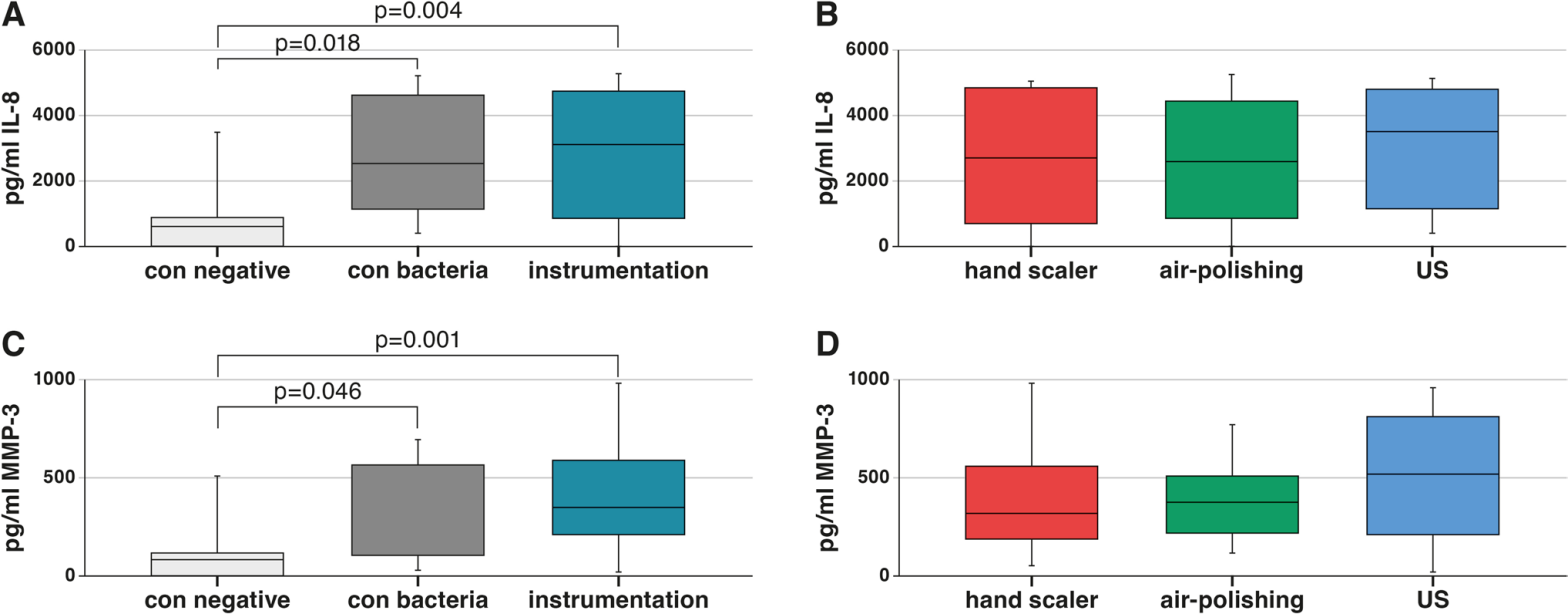
The levels of interleukin (IL)−8 (**A**, **B**) and matrix metalloprotease (MMP)−3 (**C**, **D**) in the supernatants of pulp cells (fibroblasts) seeded in the pulp chambers for 48 h were measured after incubating the teeth in a 6-species bacterial mixture for 10 weeks. Figures 4A and C compare instrumented and non-instrumented teeth with bacteria, including a negative control (con negative: no instrumentation, no bacteria), while Figs. 4B and D compare the different instrumentation methods

After 10 weeks of incubation with bacteria, pulpal cells were seeded into the pulpal chambers of the extracted teeth. Non-instrumented teeth with and without bacterial contamination served as negative controls. Median levels of IL-8 were higher in the bacterially contaminated groups, regardless of instrumentation (*p* = 0.004) or not (*p* = 0.018; both vs. control without bacterial contamination) (Fig. 5A). Similarly, higher levels of MMP-3 were measured in both the contaminated non-instrumented group (*p* = 0.046) and the instrumented group (*p* = 0.001) compared to the non-contaminated control (Fig. 5C).

Among the instrumentation groups, the ultrasonic group had the highest median levels of IL-8 (Fig. 5B) and MMP-3 (Fig. 5D), but there was no significant difference between the instrumentation groups.

## Discussion

The aim of this in vitro study was to evaluate the effect of instrumentation on the subsequent colonisation of periodontitis-associated bacteria and its potential to induce an immune response in pulp cells, primarily fibroblasts. Initially, instrumentation appeared to inhibit bacterial adhesion. However, the continuous presence of bacteria on the outer root surface induced an inflammatory response in pulp cells seeded into the cavum, irrespective of whether the surface had been instrumented.

Interestingly, instrumentation initially inhibited the colonization of the bacterial mixture on the superficial dentine layer. The instrumented root surface appeared to attract fewer bacteria and did not promote their penetration into dentine. A major difference between instrumented and non-instrumented root surfaces is the alteration of the cementum due to the instrumentation, which may be underlined also by our SEM photographs/images. Cementum is enriched with various proteins, including serum albumin, hemoglobulin, lumican, and fibrinogen [14]. These proteins may support survival of bacteria in the respective tissue, e.g. hemoglobin increased the adhesion and invasion of *P. gingivalis* in keratinocytes [15], while serum albumin might serve as an energy source [16]. The presence of specific proteins, such as serum albumin or fibrinogen, in the pellicle has been positively correlated with the presence of oral bacteria in biofilm [17]. Another important aspect to consider is modified surface roughness which is known to affect bacterial adhesion. The critical threshold is 0.2 µm [18]. Our SEM photographs suggest a smooth surface of the roots exposed to ultrasonic and air polishing treatments. In a previous study by our group and using standardised planar dentine specimens, air polishing had no influence on mean surface roughness, whereas both manual and ultrasonic instrumentation did. Manual instrumentation generally increased roughness while ultrasonic instrumentation decreased the mean roughness after one application, but resulted in higher roughness after five applications [12]. Surface roughness after ultrasonic treatment is reported differently. A recent study using extracted teeth reported ultrasonic instrumentation resulted in higher surface roughness compared to manual scaling, with the highest roughness observed in the root area [19]. In contrast, others suggest that ultrasonic scalers provide smoother surfaces with minimal damage [20]. In the present study, among the different instrumentation methods, in vitro results were less favorable for ultrasonic scaling compared to manual scaling and air polishing. Specifically, at 24 h, higher cfu counts were observed in the ultrasonic group than in the manual scaler and the air polishing groups.

When instrumenting plastic teeth in a mannequin head, root surface roughness was higher with Gracey curettes compared to sonic and ultrasonic scalers. Of interest to note, operator experience (ranging from students to trained periodontitis and dental hygienists) played a role when using manual instruments but not when using the ultrasonic scaler. No time restrictions were given in this study [21]. In contrast, our protocol included strict time limits to prevent over-instrumentation. Additionally, treatment was performed exclusively by experienced periodontists which may have influenced the results obtained after manual scaling.

In previous investigations by our group, ultrasonic instrumentation was more effective than manual scaling in reducing cfu counts in biofilms [22]. However, those investigations focused on initial biofilm removal and not on colonization of instrumented root surfaces. In the present study instrumentation was performed on cleaned root surfaces and not on a preformed biofilm which might be a limitation of the study. On the other hand, the results may suggest that a dentine surface treated with ultrasonic instrumentation is modified in a way that enables pronounced bacterial colonisation.

Regarding instrumentation modalities, systematic reviews of clinical investigations in patients with periodontitis have demonstrated that both modalities, manual instrumentation and ultrasonic instruments yield similar clinical results [23, 24].

In the long-term experiments, when teeth were incubated in culture media with bacteria for 10 weeks, the cfu counts were lower than those observed in the short-term experiments. This decline of the numbers in the long-term results might be partly explained by limited access of the bacteria to nutrients although a frequent exchange of the media was performed.

In terms of dentine penetration, a trend toward higher cfu counts was observed in the instrumented groups after 10 weeks, albeit not different among the instrumentation groups. These findings partially confirm our recent study, in which we saw a higher penetration of bacteria for scaled teeth compared to untreated controls. However, the previous investigation used two different two-species mixtures – *A. oris* and *S. gordonii,* or of *P. gingivalis* and *S. gordonii* [13] – and applied only one instrumentation modality i.e. scaling and root planing with hand curettes. Similarly, Adriaens et al. [25] analysed 21 extracted, caries-free human teeth with extensive periodontal attachment loss using light and scanning electron microscopy. They found a high penetration of bacteria into the cementum but no significant difference between treated and untreated teeth regarding the presence of bacteria in dentine. Interestingly, there was no bacterial penetration into dentine when the tooth surface was protected by attachment with Sharpey's fibers connecting the cementum to the alveolar bone. Moreover, our previous in vitro study demonstrated a negative effect of bacteria, even when killed by UV irradiation, on the attachment of epithelial cells [26]. These findings highlight the importance of biofilm removal for cell attachment and that reattachment after periodontal therapy may prevent bacterial invasion into dentine.

The close connection between the two compartments periodontium and endodontium becomes most evident in endo-periodontal lesions or in hypersensitivity reactions that may occur following instrumentation. Periodontal pathogens, in particular *T. forsythia* and *P. gingivalis*, have been detected in the endodontic tissues of most teeth presenting with endodontic-periodontal lesions [27].

In a study with monkeys, Bergenholtz and Lindhe reported pulpal alterations in 57% of teeth that were exposed to periodontal breakdown and instrumentation, compared to periodontally healthy teeth [33]. Ricucci et al. investigated the pulpal response related to the severity of periodontal disease in 48 extracted teeth [34]. Interestingly, a pulpal response was observed in teeth lacking their cementum coverage or with attachment loss extending to the apex. In the latter condition, the pulpal inflammatory response was severe, while the absence of cementum led to only minor changes. This may underline that while the cementum plays a minor role, successful periodontal therapy leading to reattachment may help maintaining endodontic health.

The pulp consists of mesenchymal connective tissue and is located within the coronal pulp chamber and the root canal extending to the apex [6]. It is lined with odontoblasts, which secrete dentine but also serve as first shield against invading bacterial compounds [6]. Moreover, the pulp tissue is composed of blood vessels, nerve fibers, immune cells, and fibroblasts [6]. In the present study, however, it can be assumed that the pulp cells seeded into the pulp were mainly pulp fibroblasts. Pulp fibroblasts are the most abundant cell type in the pulp; they play a critical role in the synthesis and replacement of extracellular matrix proteins, as well as in innate immune response and dentine-pulp regeneration [28]. Several in vitro studies have investigated the response of pulp fibroblasts to *Streptococcus mutans* and also *P. gingivalis*. Viable bacteria stimulated the expression of inflammatory cytokines such as IL-8, IL-6, or monocyte chemoattractant protein (MCP)−1 [29]. Stimulation of pulp fibroblasts by lipoteichoic acid, a component of the Gram-positive bacterial cell wall, activated the complement system targeting *S. mutans* [30]. A toxicity of *P. gingivalis* extracts on pulp cells was reported as early as 30 years ago [31]. Furthermore, *P. gingivalis* has been shown to stimulate mRNA expression of IL-8 in dental pulp fibroblasts [32]. Extracts of both *S. mutans* and *P. gingivalis* were also found to stimulate the differentiation of human pulp-cell-derived cells [36].

In the studies mentioned above, bacteria or their components were added to pulp cells cultured in well plates. In the present study, despite certain limitations (endodontic prepared teeth, pulp cells placed after ten weeks), we aimed to mimic the penetration of bacteria and their constituents, including metabolites, through the dentine barrier and to assess the effects of periodontal instrumentation. An increased expression of IL-8 and MMP-3 in pulpal cells was observed when teeth were exposed to bacteria in comparison to unexposed controls. However, no significant differences were found either between treatment per se vs. non-treated teeth or among the different treatment modalities. These findings may corroborate our previous results, which demonstrated increased IL-8, MCP-1, and MMP-3 expression in pulpal cells seeded into pulpal cava of teeth exposed to *P. gingivalis/S. gordonii*. Notably, this increase was not seen when the exposure involved *A. oris/S. gordonii* [13]. Given that the multi-species mixture of the biofilm used in the present study contained all the bacterial species investigated previously, the results may support a special role for periodontal pathobionts and further underline the importance of biofilm removal.

In this study we used extracted, caries-free premolar and molar teeth, and instrumentation of the root surfaces was performed in a standardised manner. A direct effect of treatment (whether mechanical or due to the used powder) on pulpal cells can be excluded, as the cells were seeded into the pulpal cava at a later stage. Although surface modification due to treatment may affect the penetration of bacteria and their compounds, no significant differences were found. In contrast, other in vitro research that directly exposed fibroblasts to air polishing powders reported potential effects of powders on cytokine expression, wound healing, and cell proliferation [37].

The design of the present study was complex, involving long-lasting incubation periods with frequent media changes. It included bacterial aspects at different time points, and the interaction with pulp cells (mainly fibroblasts) in extracted teeth. However, the model could not fully replicate the complexity of in vivo conditions. Addition of bacteria occurred after instrumentation, The pulp cells were placed into the pulp cavum after a ten-week bacterial incubation and then only for 48 h. As the pulp cells primarily consisted of pulpal fibroblasts, they were unable to counteract the bacterial challenges from the onset of contamination.

Nevertheless, the model allowed for the identification of differences between treated and non-treated teeth and, despite of all its limitations, to conclude on treatment strategies. The tooth surfaces underwent standardized treatment under in vitro conditions, using a defined multi-species mixture containing periodontitis-associated bacteria applied to the outer root surface.

To conclude, the data of our in vitro study suggest that colonisation of dentine with periodontitis-associated bacteria may provoke an immune response in pulp cells. Therefore, frequent instrumentation of the root surface is necessary to remove the biofilm and to support reattachment.

## Data Availability

Raw data can be obtained from the corresponding author upon request.
